# Alpha-ketoglutarate rescues impaired endothelial progenitor cell-mediated angiogenesis in diabetic mice

**DOI:** 10.3389/fphar.2025.1656473

**Published:** 2025-10-17

**Authors:** Jing-Hui Qiu, Xiao-Bao Ruan, Yu Jiang, Wen-Ting Shi, Xia Tao, Alex F. Chen, Cheng Peng, He-Hui Xie

**Affiliations:** ^1^ College of Public Health and Hongqiao International Institute of Medicine, Shanghai Jiao Tong University School of Medicine, Shanghai, China; ^2^ Department of Pharmacy, Second Affiliated Hospital of Naval Medical University, Shanghai, China; ^3^ Institute for Developmental and Regenerative Cardiovascular Medicine, Xinhua Hospital, Shanghai Jiao Tong University School of Medicine, Shanghai, China

**Keywords:** diabetes, alpha-ketoglutarate, endothelial progenitor cells, angiogenesis, cerebral ischemic injury

## Abstract

It is of great clinical significance to develop potential novel strategies to prevent diabetic cardiovascular complications. Endothelial progenitor cells (EPCs) dysfunction plays a critical role in the development of diabetic vascular complications. In the present study, we evaluated whether alpha-ketoglutarate (AKG) could improve the impaired function of EPCs, rescue EPC-mediated angiogenesis, and prevent cerebral ischemic injury in diabetic mice (*Mus musculus*). Diabetes was induced in mice by five consecutive injections of streptozotocin (STZ, 60 mg·kg^−1^·d^−1^, i. p.). The diabetic mice were randomly divided into two groups, half of the mice were treated daily by oral gavage with AKG (4 g·kg^−1^·d^−1^), and the other half were treated daily with the same amount of vehicle (saline solution) via gavage for 4 consecutive weeks. We found that administration of AKG significantly reduced the cerebral ischemic injury, promoted angiogenesis and improved EPCs function in diabetic mice. In mice just after middle cerebral artery occlusion, intravenous injection of AKG-treated diabetic EPCs displayed a greater ability to promote local angiogenesis and reduce cerebral ischemic injury compared to injection of diabetic EPCs treated with vehicle. Furthermore, we found that AKG significantly increased the expression of manganese superoxide dismutase (MnSOD) and copper-zinc SOD (CuZnSOD), decreased intracellular O_2_^·-^ levels, and attenuated inflammation in EPCs of diabetic mice. In cultured human umbilical vein endothelial cells (*Homo sapiens*, HUVECs), AKG (0.5 mM) rescued the functions of high glucose-stimulated HUVECs by reducing inflammation through the toll-like receptor 4 (TLR4)/nuclear factor kB (NF-κB) pathway and attenuating oxidative stress. In conclusion, AKG can enhance EPCs’ angiogenic potential and protect against cerebral ischemic injury in diabetic mice. It is implied that chronic treatment with AKG may be a safe and promising option to prevent ischemic diseases (including stroke) in diabetes.

## 1 Introduction

Over 589 million people worldwide are impacted by diabetes, which continues to rise in prevalence (IDF Diabetes Atlas, 2025). Cardiovascular disease (CVD) remains the leading cause of death in people with diabetes, and the risk of CVD for adults with diabetes is at least two to four times the risk in adults without diabetes (Snell-Bergeon and Wadwa, 2012). However, recent randomized controlled trials (the ACCORD and ADVANCE trials) demonstrated that, as compared with standard therapy, the use of intensive therapy to target near-normal glycemic control for a median of 3.5–5 years does not significantly reduce cardiovascular events within that time frame, but increased mortality in the ACCORD trial (Dluhy and McMahon, 2008; Miller et al., 2008; Patel et al., 2008; Sniderman et al., 2019). Thus, it should be of great clinical importance to develop new therapeutic strategies to prevent diabetic cardiovascular complications.

Diabetic cardiovascular complications are frequently associated with EPCs dysfunction and reduced EPC-mediated angiogenesis in response to ischemia (Loomans et al., 2004; Tepper et al., 2002). EPCs, the precursor of mature endothelial cells (ECs), actively participate in endothelial repair, by moving to the vascular injury site to form mature ECs and new blood vessels (Omar et al., 2010). Thus, it is postulated that promoting EPCs function and EPC-mediated ischemic angiogenesis may serve as a potential strategy to prevent cardiovascular complications (such as stroke) in diabetes.

AKG is a crucial intermediate of the Krebs cycle and plays an important role in multiple metabolic processes as well as dietary supplementation to improve health in human (Gyanwal et al., 2022). AKG promotes cardiomyocyte proliferation and heart regeneration after myocardial infarction (Sugiura et al., 2008). Previous studies have demonstrated that AKG exhibits a variety of pharmacological effects such as antioxidation and anti-inflammation (Niemiec et al., 2011; Liu et al., 2018; Tian et al., 2021; Asadi Shahmirzadi et al., 2020; He et al., 2017; Liu et al., 2023). And excessive production of oxidative stress and inflammation under diabetic conditions may impair EPC functions and disrupt its angiogenesis function (Giacco and Brownlee, 2010; Ighodaro, 2018; Donath et al., 2013; Zdzisińska et al., 2017; Bayliak and Lushchak, 2021). Based on these findings, we hypothesized that AKG may serve as a safe and effective option to prevent ischemic diseases by rescuing impaired EPC-mediated angiogenesis in diabetic mice. Here, we demonstrated that AKG could enhance EPCs’ angiogenic potential and protect against cerebral ischemic injury in diabetic mice.

## 2 Materials and methods

### 2.1 Animal models of diabetes and AKG treatment

Male C57BL/6 mice at 10–12 weeks of age (25–30 g, purchased from the Sino-British SIPPR/BK Laboratory Animal Co., Ltd.) were rendered diabetic by intraperitoneal (i.p.) injection of 60 mg/kg STZ (Sigma-Aldrich, St Louis, MO, United States) in 0.05 M sodium citrate (pH 4.5) daily for five consecutive days during the first week of the study (Zauli et al., 2010; Wang et al., 2011). Previous studies reported that estrogens exert neuroprotective effects in an animal model of ischemia (Suzuki et al., 2009; Shi et al., 2024). To avoid the interference of estrogen on ischemic stroke, only male mice were selected in this study. Fasting blood glucose levels were measured from tail blood samples using a OneTouch glucose meter (LifeScan), starting from the initiation of streptozotocin (STZ) injection and monitored weekly thereafter. A 5-day low-dose STZ injection regimen was used to ensure sustained hyperglycemia (Wang et al., 2011). After 14 days, whole fasting blood glucose levels from tail blood were measured, and mice with a blood glucose level>11.1 mmol/L were considered diabetic (Ju et al., 2018). The diabetic mice were randomly divided into two groups, half of the mice were treated daily by oral gavage with AKG (4 g·kg^−1^·d^−1^; Sigma-Aldrich, St Louis, MO, United States), and the other half were treated daily with the same amount of vehicle (saline solution) via gavage for 4 consecutive weeks. To ensure consistency in AKG intake among mice, we conducted a preliminary experiment to measure water intake. Based on daily water consumption, AKG at 2% in drinking water corresponds to doses of 4 g·kg^−1^·d^−1^ (Yuan et al., 2022; Cheng et al., 2024; An et al., 2021). After 4 weeks of AKG (Sigma-Aldrich) treatment, mice were used for EPCs isolation and assessment, or were subjected to permanent focal cerebral ischemia. Experiments were carried out in a random and blinded fashion. Experimental procedures were in accordance with the National Institutes of Health Guide for the Care and Use of Laboratory Animals (United States). All animal experiments complied with the ARRIVE guidelines. The animal protocol was planned in compliance with the animal protection, animal welfare, and ethical principles and was approved by the IACUC of the Shanghai Jiao Tong University.

### 2.2 Animal model of stroke

Permanent focal cerebral ischemia was induced in mice according to previously described methods (Dong et al., 2017; Dong et al., 2015). Briefly, mice were anesthetized with 3.5% chloral hydrate at 0.1 mL/10 g body weight by intraperitoneal injection. A 0.5-cm skin incision was made between the left orbit and ear. Then, the left distal middle cerebral artery was exposed through a craniotomy and permanently cauterized above the rhinal fissure. The body temperature was maintained at 37 °C ± 0.5 °C throughout the surgical procedure using a heating lamp and pad. Behavioral assessment (including Body Asymmetry Test and Beam Test) was performed 3 days after middle cerebral artery occlusion, then mice were euthanized, and their brains were immediately removed. Infarct volume was determined by staining with 2, 3, 5-triphenyltetrazolium chloride (TTC, Sinopharm Chemical Reagent Co., Ltd., Shanghai, China) and was analyzed with ImageJ software (Dong et al., 2017; Zhu et al., 2008). The experiments were performed in a blind and random fashion.

Additionally, on day 3 after middle cerebral artery occlusion, the mice were anesthetized and the ischemic brains were flushed with PBS and perfused in 4% PFA, before being dehydrated and embedded in paraffin. Serial sections (6 µm thick) were cut in the coronal plane from paraffin-embedded blocks. Every 10th section was processed for immunohistochemical staining. For quantification of angiogenesis, the area of ischemic boundary was immunostained with CD31 and von Willebrand Factor (vWF) antibody (Dong et al., 2017; Zhang et al., 2002; Chen et al., 2005).

### 2.3 Neurological behavior analysis: body asymmetry test and beam test

Body Asymmetry Test—To measure motor asymmetry, the mice were examined using the elevated body swing test as described previously (Dong et al., 2017; Dong et al., 2015). The mice were examined for head swings while being suspended by their tails. The direction of the swing (right or left) was recorded when the mouse turned its head sideways by approximately at a 10° angle to the body’s midline. After each swing, each mouse was allowed to move freely in a Plexiglas box for at least 30 s before undergoing the next test; the trials were repeated 20 times for each animal. The frequency of the head swings toward the contralateral side was counted and normalized as follows: (number of lateral swings in 20 tests- 10)/10 %× 100%.

Beam Test—Beam walking across a bridge was used to assess motor coordination and balance after stroke injury. The mice were trained for 5 days before the MCA occlusion to traverse a narrow round beam (5 mm in diameter, 900 mm in length) to reach an enclosed escape platform. They were placed on one end of the beam and the latency to traverse the central 80% of the beam toward the enclosed escape platform at the other end was recorded. Data are expressed as mean latency to cross the beam in three trials (Dong et al., 2017; Dong et al., 2015).

### 2.4 Bone marrow-derived EPCs (BM-EPCs) isolation, culture and characterization

BM-EPCs were isolated and cultured according to the published methods (Xie et al., 2010; Peng et al., 2018). Bone marrow-derived mononuclear cells (BMMNCs) were isolated from the mice tibia and femur, seeded in six-well cell culture plates coated with rat vitronectin (1 mg/mL; Sigma-Aldrich, St Louis, MO, United States) at a density of 5 × 10^6^ cells/well, and cultured in endothelial growth medium-2 (EGM-2; Lonza, Walkersville, MD, United States). After 4 days of culture, nonadherent cells were removed and the adherent cells were further cultivated for 3 days. And then EPCs were either used for *in vitro* studies (including function assays and Western blot analysis).

The EPCs were characterized via flow cytometry for the co-expression of Sca-1 and Flk-1, and through Dil-acLDL/lectin/Hoechst triple staining, following our previously described methodologies (Xie et al., 2010; Peng et al., 2018). After a 7-day culture, the attached cells were labeled with Dil-acLDL (10 μg/mL; Invitrogen) at 37 °C for 4-h, washed three times in PBS, and immersed in 2% paraformaldehyde for 10 min. The cells were then incubated with FITC-labeled Ulexeuropaeus agglutinin (lectin, 10 μg/mL; Sigma–Aldrich) for 1-h. After nuclei staining with a Hoechst stain (5 μg/mL; Sigma–Aldrich), the cells were viewed under an inverted fluorescent microscope (Leica). Pictures were taken of high-power fields (magnification, ×200). Cells demonstrating triple-positive fluorescence of Dil-acLDL, lectin, and the Hoechst dye were identified as EPCs (Xie et al., 2010; Peng et al., 2018) (data not shown). The phenotype of the EPCs was further examined by flow cytometry. EPCs were placed in polypropylene tubes in 100 μL PBS with 1% albumin. They were then stained with FITC-conjugated Sca-1 antibodies (BD Biosciences) and PE-conjugated Flk-1 antibodies (BD Biosciences) for 1 h at 4 °C and washed three times in PBS with 1% albumin. Quantitations of Sca-1+/Flk-1+ cells were performed with a BD Flow cytometer (Xie et al., 2010; Peng et al., 2018) (data not shown).

### 2.5 EPCs adhesion assay

1 × 10^4^ cells were plated per well of a 96-well plates precoated with vitronectin (1 μg/mL; Sigma-Aldrich, St Louis, MO, United States). After 4-h incubation time, nonadherent BM-EPCs were removed, and residual adherent BM-EPCs were stained with 10 μg/mL of Hoechst (Hoechst 33,258; Sigma-Aldrich, St Louis, MO, United States) and then fixed with 2% PFA. Adherent cells were counted at five random fields at magnification of ×100 per sample. Three wells were measured for each cell sample (Dong et al., 2017).

### 2.6 EPCs migration assay

BM-EPCs were plated at a number of 5 × 10^4^ per well in the upper Boyden’s chamber with M199 (Hyclone, Shanghai, China), while M199 supplemented with 50 ng/mL of VEGF (R&D systems, Minneapolis, MN, United States) and 10% FBS (Bioind, Kibbutz Beit Haemek, Israel) was placed in the lower chamber. After 24-h incubation time, migrated BM-EPCs adhering to the lower membrane were fixed and then stained with 10 μg/mL of Hoechst 33,258 (SigmaAldrich). Then, the number of migrated BM-EPCs were determined by counting the BM-EPCs on the lower side of the membrane under a microscope at magnification ×100. In blinded analyses, for each sample, five images were taken from random fields (Dong et al., 2017).

### 2.7 EPCs tube formation assay

A number of 5 × 10^4^ BM-EPCs were plated per well of a 96-well plate precoated with growth factor-reduced Matrigel-Matrix (BD Biosciences, San Jose, CA, United States). After 6-h of incubation, images of tube morphology were taken by inverted microscope (Leica), and tube numbers were counted at five random fields at magnification of ×100 per sample (Dong et al., 2017; Marrotte et al., 2010).

### 2.8 BM-EPCs intracellular superoxide (O_2_^·-^) measurement

O_2_^·-^ levels were estimated using the fluorescent probe dihydroethidium (DHE) (Sigma-Aldrich, St Louis, MO, United States), a commonly used cell-permeable dye which is sensitive to O_2_^·-^ and may be oxidized to the red fluorescent molecule ethidium. BM-EPCs were washed, collected and resuspended in M199 medium, and then labeled with 1 µM DHE for 40-min at 37 °C in dark. After that, labeled BM-EPCs were washed three times with 5% BSA/PBS and then fixed in 2% PFA. Finally, the labeled BM-EPCs were analyzed by flow cytometry (Xie et al., 2010).

### 2.9 EPCs transplantation and animal stroke model in diabetic mice

To further confirm whether the EPC-mediated ischemic angiogenesis might relate to the protection of AKG against cerebral ischemic injury in diabetic mice, a total of 1 × 10^6^ bone marrowderived diabetic EPCs and AKG-treated diabetic EPCs in 200 μL PBS were respectively systemically injected into diabetic mice via a tail vein just after middle cerebral artery occlusion, and equivalent volume of PBS was administered to control diabetic mice (Dong et al., 2015). On day 3 after stroke, behavioral tests were performed, and then the ischemic brains were serially cut and stained with 2% TTC for 5-min (55 °C) to determine the infarct area (Dong et al., 2017; Zhu et al., 2008).

Additionally, on day 3 after middle cerebral artery occlusion, the mice were anesthetized and the ischemic brains were flushed with PBS and perfused in 4% PFA, before being dehydrated and embedded in paraffin. Serial sections (6 µm thick) were cut in the coronal plane from paraffin-embedded blocks. Every 10th section was processed for immunohistochemical staining. For quantification of angiogenesis, the area of ischemic boundary was immunostained with CD31 and vWF antibody (Dong et al., 2017; Zhang et al., 2002; Chen et al., 2005).

### 2.10 HUVECs culture and treatment

To check whether the same results observed in EPCs could be obtained in human endothelial cells, we repeated these experiments on HUVECs. HUVECs were purchased from the Chinese Academy of Sciences (Shanghai, China). Cells were cultured in RAPI 1640 medium (Gibco, America) supplemented with 10% foetal bovine serum (FBS) at 37 °C in a CO_2_ incubator according to the manufacturer’s instructions. HUVECs were seeded in 6-well plates at a density of 1 × 10^5^ cells/mL to reach 80%–90% confluence. The cell number was manually counted in a hemocytometer. Cells were fasted overnight in serum-free 1,640 medium followed by incubation for 24-h with mannitol (33 mM) or high glucose (HG, 33 mM) in the absence or presence of α-Ketoglutanc acid disodium salt dihydrate (Sigma, 75,892). The glucose level (33 mM) used in the present work was determined according to previous studies (Thum et al., 2007; Eriksson et al., 2012; Xue et al., 2008).

### 2.11 HUVECs viability assay

To examine the cytotoxicity of AKG, the viability of cells after treatment with different doses of AKG were assessed via using Cell Counting Kit-8 (CCK-8, Beyotime, Shanghai, China). The cells were cultured in a 96-well plate at a density of 10,000 cells/well and incubated for 24-h. When the cells were to 90% confluence, AKG was added to different groups at final concentrations of 0.05, 0.1, 0.5, 1, 2, or 8 mM. After 24-h, the original medium was replaced with 100 μL medium containing 10% (v/v) CCK-8 and incubated with an additional 1-h. The optical density (OD) values at 450 nm were measured using a microplate reader (Yang et al., 2023).

### 2.12 HUVECs adhesion assay

1 × 10^4^ cells were plated per well of a 96-well plates precoated with vitronectin (1 μg/mL; Sigma-Aldrich, St Louis, MO, United States). After 4-h incubation time, nonadherent HUVECs were removed, were removed, and residual adherent HUVECs were stained with 10 μg/mL of Hoechst (Hoechst 33,258; Sigma-Aldrich, St Louis, MO, United States) and then fixed with 2% PFA. Adherent cells were counted at five random fields at magnification of ×100 per sample. Three wells were measured for each cell sample (Dong et al., 2017).

### 2.13 HUVECs migration assay

HUVECs were plated at a number of 5 × 10^4^ per well in the upper Boyden’s chamber with M199 (Hyclone, Shanghai, China), while M199 supplemented with 50 ng/mL of VEGF (R&D systems, Minneapolis, MN, United States) and 10% FBS (Bioind, Kibbutz Beit Haemek, Israel) was placed in the lower chamber. After 24-h incubation time, migrated HUVECs adhering to the lower membrane were fixed and then stained with 10 μg/mL of Hoechst 33,258 (SigmaAldrich). Then, the number of migrated HUVECs were determined by counting the HUVECs on the lower side of the membrane under a microscope at magnification ×100. In blinded analyses, for each sample, five images were taken from random fields (Dong et al., 2017).

### 2.14 HUVECs intracellular superoxide (O_2_^·-^) measurement

Intracellular superoxide (O_2_^·-^) level was determined using DHE, a membrane-permeable dye which is oxidized to ethidium bromide in the presence of O_2_^·-^ (Marrotte et al., 2010; Guerby et al., 2019). After treatment with or without 0.5 mM AKG for 24-h in RPMI 1640 with high glucose, HUVECs were trypsinized and rinsed with RPMI 1640, and then incubated with DHE (10–6 mol/L) for 30-min at 37 °C in dark. After incubation, cells were washed with 5% BSA (w/v) in PBS and re-suspended in 300 μL 2% paraformaldehyde. The DHE fluorescence intensity in cells was determined by flow cytometry.

### 2.15 Western blot analysis

Western blot analysis was performed as previously described (Zhang et al., 2002). Briefly, BM-EPCs were collected by trypsinization and washed with PBS. Total protein lysates were prepared in lysis buffer containing protease inhibitors (KC-440, KangChen), and centrifuged at 12,000 *g* for 10-min at 4 °C. Protein concentrations were determined using the Pierce BCA assay kit (Thermo Fisher), and samples containing equal amounts of protein were subjected to 10% (v/v) SDS/PAGE. Proteins were transferred to nitrocellulose membranes, blocked with 5% (w/v) non-fat dry milk (Beyotime) and incubated overnight at 4 °C with the following primary antibodies: MnSOD (1:1,000), CuZnSOD (1:500), TLR4 (1:1,000), NF-κB (1:600), IL-6 (1:1,000), IL-1β (1:1,000) and β-actin (1:5,000). Secondary antibodies were IRDye^®^ 800CW Goat anti-Rabbit Antibody (1:10,000) and Goat anti-Mouse Antibody (1:5,000). Bands were visualized using Odyssey Imager with Odyssey 1.1 software (Li-Cor) and quantified using NIH image J software.

HUVECs were collected by trypsinization and washed with PBS after treatment with or without 0.5 mM AKG for 24-h in RPMI 1640 with high glucose. Total protein lysates were prepared in lysis buffer containing protease inhibitors (KC-440, KangChen), and centrifuged at 12,000 *g* for 10 min at 4 °C. Protein concentrations were determined using the Pierce BCA assay kit (Thermo Fisher), and samples containing equal amounts of protein were subjected to 10% (v/v) SDS/PAGE. Proteins were transferred to nitrocellulose membranes, blocked with 5% (w/v) non-fat dry milk (Beyotime) and incubated overnight at 4 °C with the following primary antibodies: MnSOD (1:1,000), CuZnSOD (1:500), TLR4 (1:1,000), p-NF-κB (1:600), NF-κB (1:600), TNF-α (1:500), IL-6 (1:1,000), IL-1β (1:1,000) and β-actin (1:5,000). Secondary antibodies were IRDye^®^ 800CW Goat anti-Rabbit Antibody (1:10,000) and Goat anti-Mouse Antibody (1:5,000). Bands were visualized using Odyssey Imager with Odyssey 1.1 software (Li-Cor) and quantified using NIH image J software.

### 2.16 Pharmacological interventions in HUVECs

CLI-095 is a selective TLR4 antagonist that blocks the TLR4 signaling pathway in mice (Zhang et al., 2022). MD2-TLR4-IN-1 inhibits TLR4 activation by binding to its co-receptor MD2, thereby preventing ligand-induced (e.g., LPS) TLR4 signaling (Liu et al., 2019). JSH-23 selectively inhibits NF-κB by preventing nuclear translocation of the p65 subunit, thus suppressing transcription of NF-κB target genes (Shin et al., 2004; Kumar et al., 2011). To further examine whether AKG exerted anti-inflammatory roles by inhibiting the TLR4/NF-κB pathways, antibodies against TLR4 intracellular binding domain (CLI-095), extracellular binding domain (MD2-TLR4-IN-1) and a selective NF-κB inhibitor (JSH-23) were added at final concentrations of 10 μmol/L. Briefly, HUVECs were pretreated with or without CLI-095, MD2-TLR-4-IN-1 or JSH-23 for 1-h before incubation with or without 0.5 mM AKG for 24-h in RPMI 1640 with high glucose for 24-h (Janovec et al., 2021; Oh et al., 2022). Then, the cells were harvested by trypsin digestion and centrifugation for Western blotting analysis.

### 2.17 Statistical analysis

Data were expressed as mean ± SEM. One-way ANOVA followed by Newman-Keuls *post hoc* analysis was used for comparison of variables in more than two groups. A value of P < 0.05 was considered statistically significant.

## 3 Results

### 3.1 AKG had no effect on fasting blood glucose levels and body weights in diabetic mice

**FIGURE 1:**
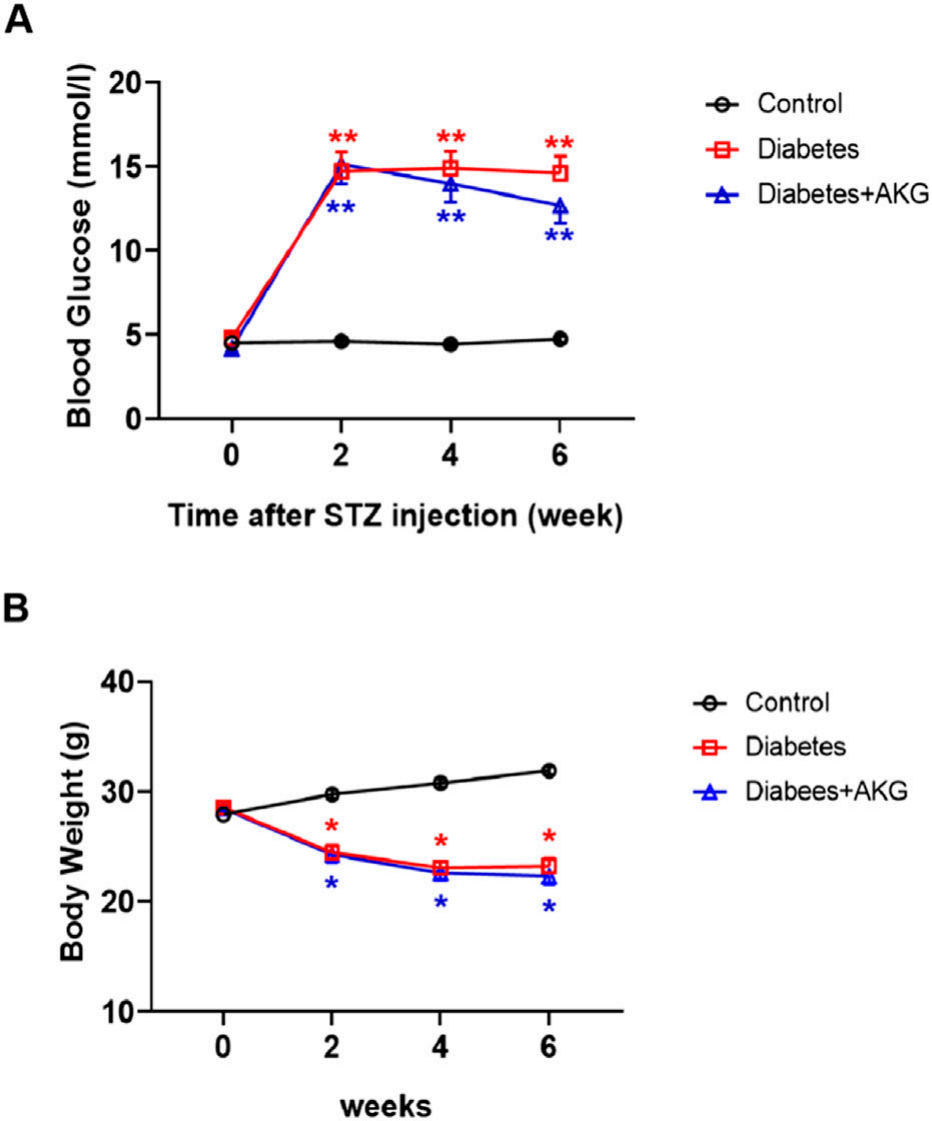
Effects of AKG treatment on fasting blood glucose levels and body weights in diabetic mice. **(A)**, Fasting blood glucose levels. **(B)**, Body weights. Throughout, error bars represent mean ± SEM. n = 10–12 per group. *p < 0.05, **p < 0.01 vs. Control. Control, vehicle (citrate buffer and saline solution)-treated mice; Diabetes, vehicle (saline solution)-treated diabetic mice; Diabetes + AKG, alpha-ketoglutarate-treated diabetic mice.

A 5-day low-dose STZ injection regimen was used to ensure sustained hyperglycemia (Liu et al., 2018). Five consecutive intraperitoneal daily injections of STZ (60 mg·kg^−1^·d^−1^) were administered to male C57BL/6 mice, after 14 days, whole blood glucose levels were measured with a OneTouch meter (LifeScan) and mice with a blood glucose level >11.1 mmol/L were considered diabetic. Half of the diabetic mice were treated daily by oral gavage with AKG (4 g·kg^−1^·d^−1^) for four consecutive weeks, and the other half were treated daily by oral gavage with vehicle (saline solution) for four consecutive weeks. As shown in Figure 1 significant increase in fasting blood glucose levels and a considerable reduction in body weight were observed in the STZ-treated mice. However, 4 weeks treatment with AKG had no obvious effect on fasting blood glucose levels and body weight in diabetic mice.

### 3.2 AKG treatment protected diabetic mice against cerebral ischemic injury

**FIGURE 2:**
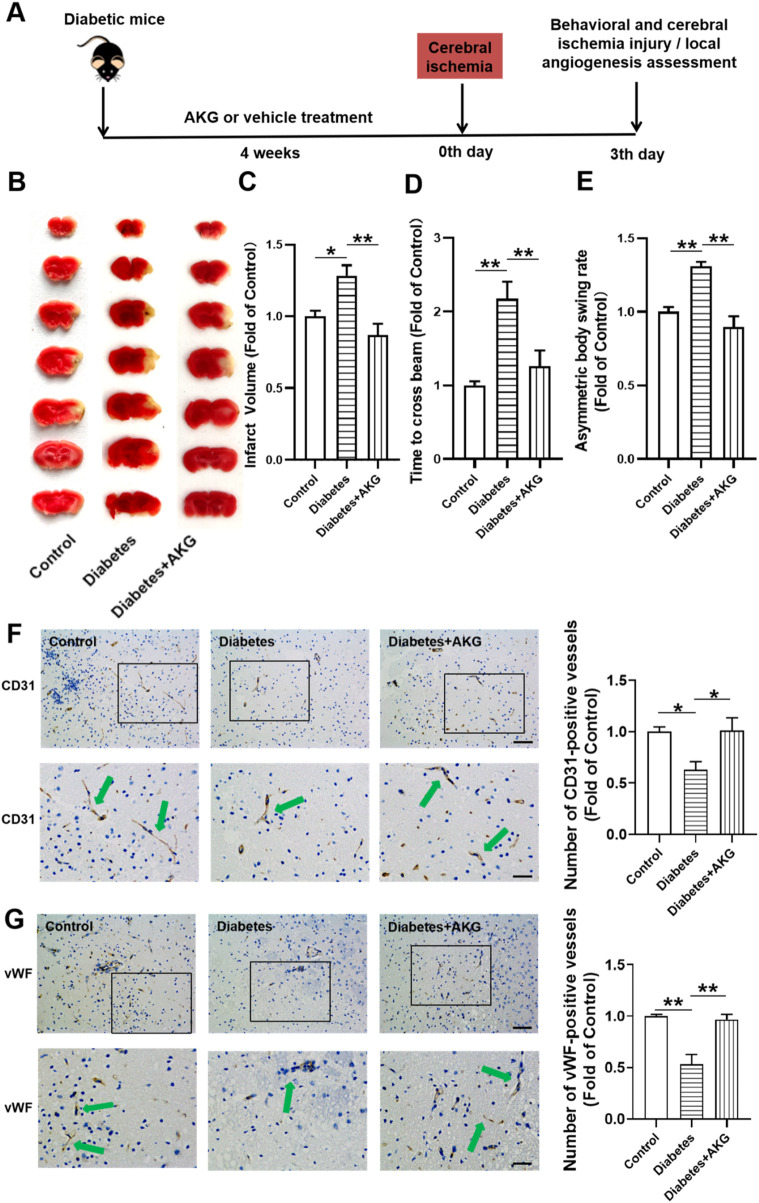
AKG promoted angiogenesis in ischemic brain and protected against cerebral ischemic injury in diabetic mice. **(A)** Experimental protocols: five consecutive intraperitoneal daily injections of STZ (60 mg/kg) were administered to male C57BL/6 mice that were monitored for 6 weeks. After 14 days, mice with a blood glucose level>11.1 mmol/L were considered diabetic. The diabetic mice were randomly divided into two groups, half of the mice were treated daily by oral gavage with alpha-ketoglutarate (4 g·kg^−1^·d^−1^), and the other half were treated daily with the same amount of vehicle (saline solution) via gavage for four consecutive weeks. The control mice were treated with intraperitoneal injection of citrate buffer and subsequently with saline solution by oral gavage. Then the mice were subjected to focal cerebral ischemia by permanent occlusion. Infarct volumes, functional outcome and the local angiogenesis in ischemic brain 3 days after focal cerebral ischemia were investigated in mice of ischemic stroke. **(B)** Images are representative of 2,3,5-triphenyltetrazolium chloride-stained ischemic brain sections, cerebral infarct volumes **(C)** and neurobehavioral outcomes (**(D)** Beam Test; **(E)** Body Asymmetry Test) on day 3 after middle cerebral artery occlusion. n = 6. Values were normalized to Control. *p < 0.05, **p < 0.01. **(F)** Microvessels (CD31^+^) in the boundary area of ischemic brains. The bar graph shows that the number of microvessels was reduced in diabetic mice compared with control, which was significantly increased in AKG treated diabetic mice. n = 6. Scale bar, 100 μm (top); 50 μm (bottom). **(G)** Microvessels (vWF) in the boundary area of ischemic brains. The bar graph shows that the number of microvessels was reduced in diabetic mice compared with control, which was significantly increased in AKG treated diabetic mice. n = 3. Scale bar, 100 μm (top); 50 μm (bottom). Control, vehicle (citrate buffer and saline solution)-treated mice; Diabetes, vehicle (saline solution)-treated diabetic mice; Diabetes + AKG, Alpha-ketoglutarate-treated diabetic mice.

After 4-week of treatment, mice were subjected to cerebral ischemia (Figure 2A). Compared to control mice, diabetic mice exhibited more severe cerebral ischemic injury (+28.3%, *p* < 0.05) and higher neurological deficits at 3 days after cerebral ischemia. However, the infarct volume was significantly reduced (−41.4%, *p* < 0.01) and the neurological deficits were improved in AKG treated diabetic mice compared with diabetic mice (Figures 2B–E). These results indicate that AKG treatment could protect diabetic mice against cerebral ischemic injury.

### 3.3 AKG treatment promoted local angiogenesis in ischemic brain in diabetic mice

Angiogenesis is the fundamental processes during formation of new blood vessels after vascular injury, following ischemic stroke (Liman and Endres, 2012). Therefore, local angiogenesis was assessed by immunostaining at 3 days after cerebral ischemia using an antibody against the angiogenesis marker, i.e., CD31 (Figure 2F). It was found that the number of CD31^+^ microvessels in the boundary area of ischemic brains was reduced in diabetic mice compared with control (−37.2%, *p* < 0.05), which was significantly increased in 4-week AKG treated diabetic mice (+38.3%, *p* < 0.05 vs. Diabetes) (Figure 2F). ECs specifically produce vWF, a marker that stains newly formed vessels (Yu et al., 2019). To confirm whether angiogenesis is activated, we evaluated another angiogenic marker vWF to validate this study. It was found that the number of vWF microvessels in the boundary area of ischemic brains was reduced in diabetic mice compared with control (−46.5%, *p* < 0.01), which was significantly increased in 4-week AKG treated diabetic mice (+42.9%, *p* < 0.01 vs. Diabetes) (Figure 2G).

### 3.4 AKG treatment rescued BM-EPCs dysfunction in diabetic mice

**FIGURE 3:**
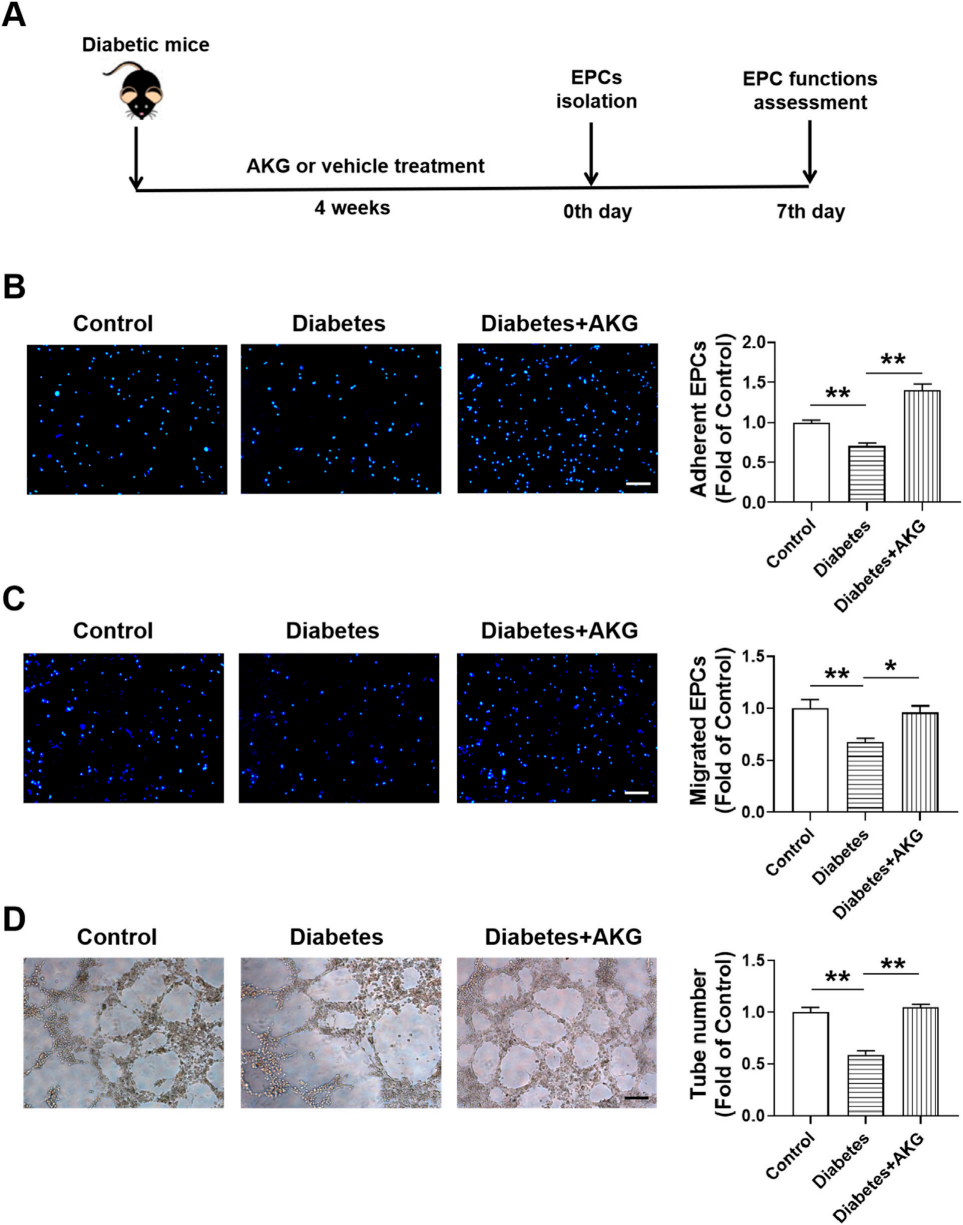
AKG treatment rescued EPCs dysfunctions in diabetic mice. **(A)** Experimental protocols: five consecutive intraperitoneal daily injections of STZ (60 mg/kg) were administered to male C57BL/6 mice that were monitored for 6 weeks. After 14 days, mice with a blood glucose level>11.1 mmol/L were considered diabetic. The diabetic mice were randomly divided into two groups, half of the mice were treated daily by oral gavage with alpha-ketoglutarate (4 g·kg^−1^·d^−1^), and the other half were treated daily with the same amount of vehicle (saline solution) via gavage for 4 consecutive weeks. The control mice were treated with intraperitoneal injection of citrate buffer and subsequently with saline solution by oral gavage. Then, BM-EPCs were isolated, cultured and examined. **(B)** EPCs adhesion assay (n = 6). **(C)** EPCs migration assay (n = 6). **(D)** EPCs tube formation assay (n = 6). Values were normalized to Control. *p < 0.05, **p < 0.01. Scale bar, 100 μm. Control, vehicle (citrate buffer and saline solution)-treated mice; Diabetes, vehicle (saline solution)-treated diabetic mice; Diabetes + AKG, Alpha-ketoglutarate-treated diabetic mice.

Diabetic cardiovascular complications are frequently associated with EPCs dysfunction and reduced EPC-mediated angiogenesis in response to ischemia (Loomans et al., 2004; Tepper et al., 2002). Therefore, after 4 weeks of treatment, the BM-EPC functions were determined in diabetic mice (Figure 3A). Results showed that the adhesion, migration and tube formation functions were significantly impaired in diabetic BM-EPCs, which were rescued by AKG treatment (*p* < 0.01, Figures 3B,D; *p* < 0.05; Figure 3C).

### 3.5 Effects of AKG administration on MnSOD, CuZnSOD and intracellular O_2_^·-^ expression levels of BM-EPCs in diabetic mice

**FIGURE 4:**
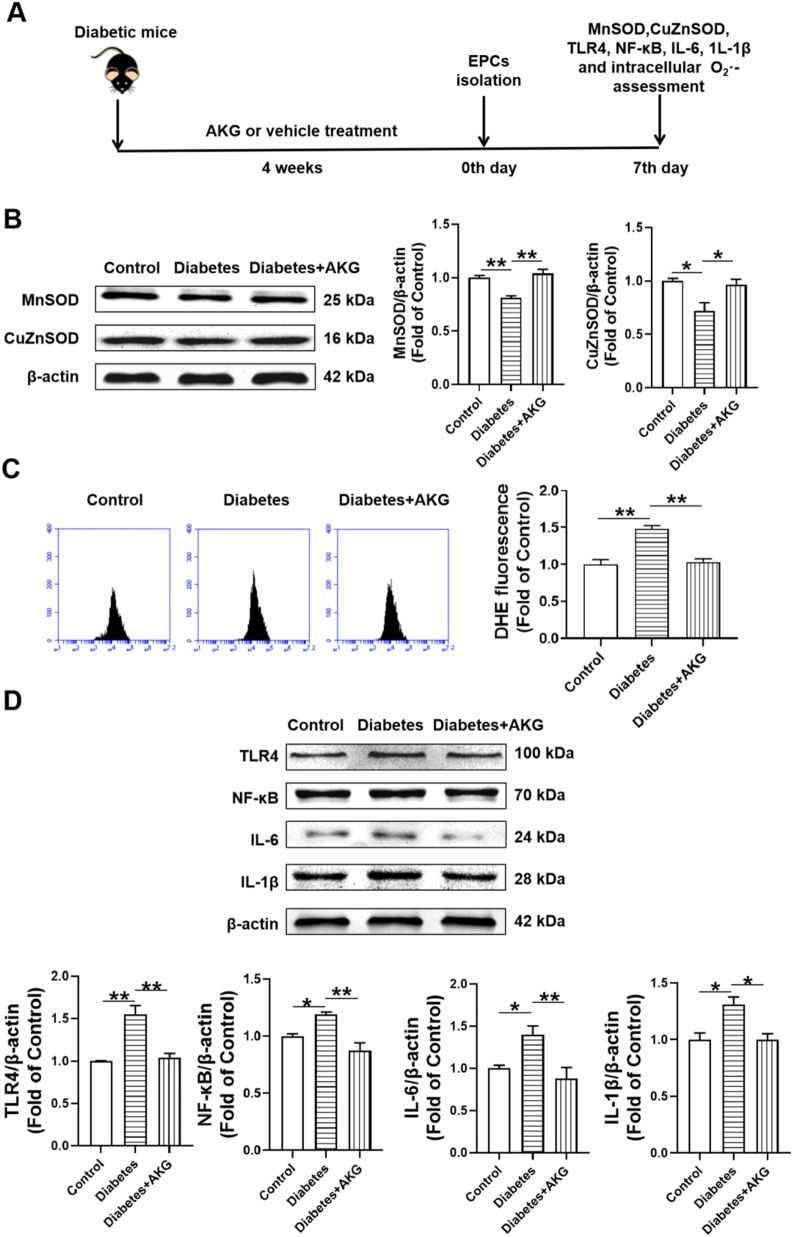
Effects of AKG administration on MnSOD, CuZnSOD, intracellular O_2_^·-^, TLR4, NF-κB, IL-6 and IL-1β expression levels of EPCs in diabetic mice. **(A)** Experimental protocols: five consecutive intraperitoneal daily injections of STZ (60 mg/kg) were administered to C57 male mice that were monitored for 6 weeks, in comparison with the group of vehicle (citrate buffer and saline solution)-treated control mice. After 14 days, mice with a blood glucose level>11.1 mmol/L were considered diabetic. The diabetic mice were randomly divided into two groups, half of the mice were treated daily by oral gavage with alpha-ketoglutarate (4 g·kg^−1^·d^−1^), and the other half were treated daily with the same amount of vehicle (saline solution) via gavage for 4 consecutive weeks. The control mice were treated with intraperitoneal injection of citrate buffer and subsequently with saline solution by oral gavage. Then, EPCs were isolated, cultured and examined. **(B)** The representative images and the protein expression levels of MnSOD (n = 3) and CuZnSOD (n = 4). **(C)** Intracellular O_2_^·-^ levels of EPCs (n = 5–6). **(D)** The representative images and the protein expression levels of TLR4 (n = 3), NF-κB (n = 3), IL-6 (n = 6) and IL-1β (n = 3). Values were normalized to Control. Values were normalized to Control. *p < 0.05, **p < 0.01. Control, vehicle (citrate buffer and saline solution)-treated mice; Diabetes, vehicle (saline solution)-treated diabetic mice; Diabetes + AKG, Alpha-ketoglutarate-treated diabetic mice.

To investigate the potential mechanisms underlying AKG protecting BM-EPCs functions, the expression of MnSOD, CuZnSOD and the levels of intracellular O_2_^·-^ were examined in diabetic mice after 4-week AKG treatment (Figure 4A). Compared to control, both MnSOD and CuZnSOD were significantly decreased (−19.2%, *p* < 0.01 and −28.1%, *p* < 0.05 respectively) in BM-EPCs from diabetic mice. BM-EPCs from AKG treatment diabetic mice showed higher levels of both MnSOD and CuZnSOD compared to diabetic mice (+23.0%, *p* < 0.01 and +24.7%, *p* < 0.05) (Figure 4B). As shown in Figure 4C, intracellular O_2_^·-^ level of BM-EPCs from diabetic mice was increased ∼50% compared to control group (+48.4%, *p* < 0.01). However, it was significantly attenuated in BM-EPCs from AKG treated diabetic mice when compared with that of the diabetic mice (−45.3%, *p* < 0.01).

### 3.6 Effects of AKG administration on TLR4, NF-κB, IL-6 and IL-1β expression levels of BM-EPCs in diabetic mice

To investigate the potential mechanisms underlying AKG protecting BM-EPCs functions, the expression of TLR4, NF-κB, IL-6 and IL-1β were examined in diabetic mice after 4-week AKG treatment (Figure 4D). Compared to control, the expression of TLR4, NF-κB, IL-6 and IL-1β were significantly increased (+35.4%, *p* < 0.01; +35.9%, *p* < 0.05; +40.1%, *p* < 0.05 and +31.1%, *p* < 0.05) in BM-EPCs from diabetic mice. BM-EPCs from AKG treatment diabetic mice showed lower levels of TLR4, NF-κB, IL-6 and IL-1β compared to diabetic mice (−32.6%, *p* < 0.01; −59.7%, *p* < 0.01; −52.5%, *p* < 0.01 and −30.9%, *p* < 0.05).

### 3.7 AKG enhanced the therapeutic effect of diabetic BM-EPCs on cerebral ischemic injury reduction and angiogenesis promotion in diabetic mice

**FIGURE 5:**
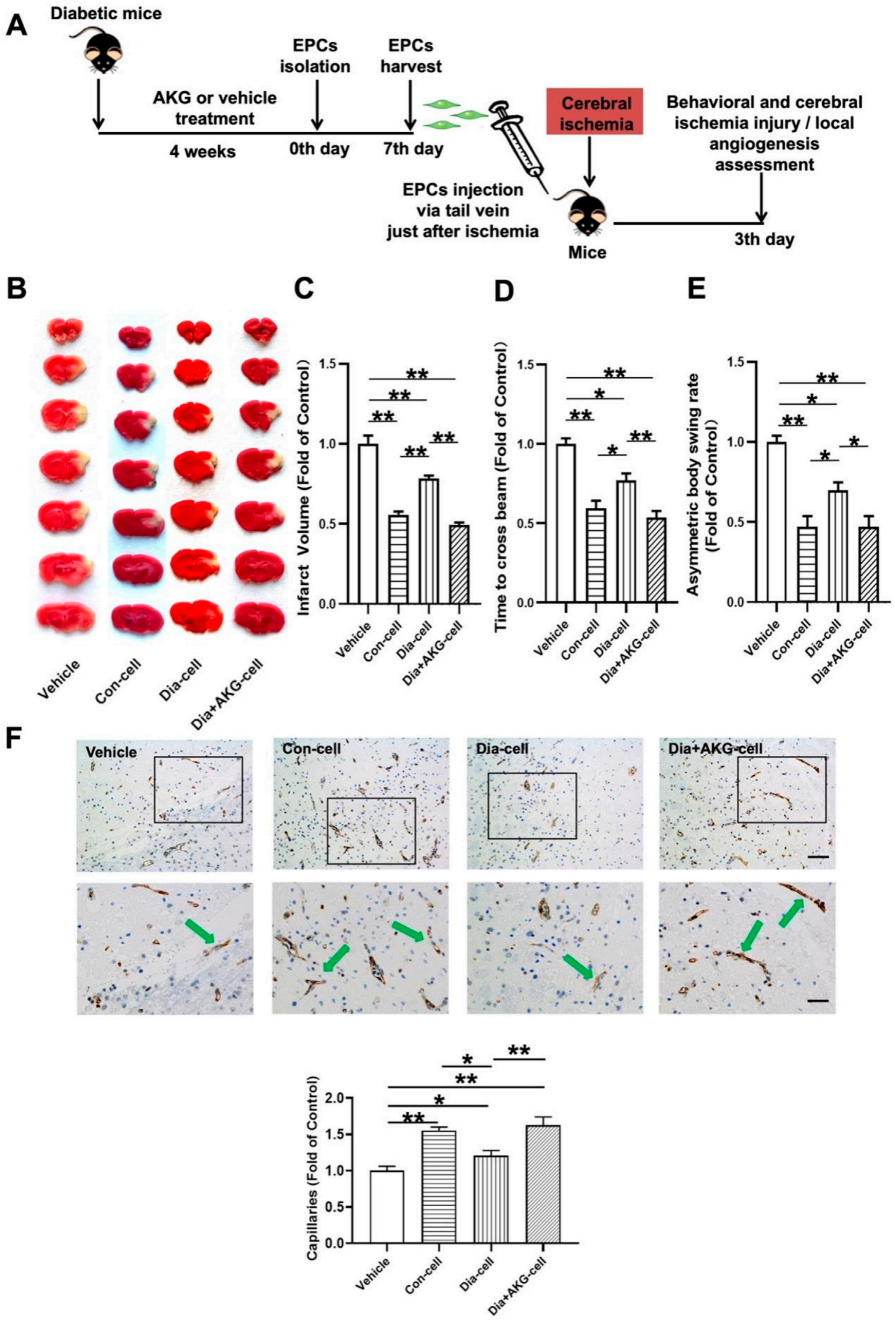
AKG improve the therapeutic effect of EPCs on cerebral ischemic injury and angiogenesis in ischemic brain in mice. **(A)** Surgical protocols: male C57BL/6 mice (10–12 weeks) were randomly allocated to 4 groups. Each group of animals respectively received a single injection of vehicle, EPCs from control mice, EPCs from diabetic mice or EPCs from diabetic mice with AKG (4 g·kg−1·d−1) treatment just after cerebral ischemia. Infarct volumes, functional outcome and the local angiogenesis in ischemic brain 3 days after focal cerebral ischemia were investigated in mice of ischemic stroke. **(B)** Images are representative of 2,3,5-triphenyltetrazolium chloride-stained ischemic brain sections, cerebral infarct volumes **(C)**, and neurobehavioral outcomes (**(D)**, Beam Test; **(E)** Body Asymmetry Test) on day 3 after middle cerebral artery occlusion. n = 7–12. Values were normalized to Control. *p < 0.05, **p < 0.01. **(F)** Microvessels (CD31^+^) in the boundary area of ischemic brains. The bar graph shows that the number of microvessels was reduced in diabetic mice compared with control, which was significantly increased in AKG treated diabetic mice. n = 6. Scale bar, 100 μm (left); 50 μm (right). Vehicle, mice treated with vehicle; Con-cell, mice treated with EPCs from control mice; Dia-cell, mice treated with EPCs from diabetic mice; Dia + AKG-cell, mice treated with EPCs from diabetic mice with AKG treatment.

To determine whether AKG can enhance the therapeutic effect of diabetic BM-EPCs on cerebral ischemic injury reduction and whether the EPC-mediated ischemic angiogenesis may contribute to the protection of AKG against cerebral ischemic injury in diabetic mice, 1 × 10^6^ bone marrow-derived EPCs from normal mice, diabetic mice and diabetes mice with AKG treated were injected via tail vein into normal mice immediately after middle cerebral artery occlusion, and control mice received equal volume of vehicle (PBS) (Figure 5A).

As shown in Figures 5B–E, normal BM-EPCs, diabetic BM-EPCs and AKG treated diabetic BM-EPCs transplantation attenuated infarct volume in mice (−44.3%, *p* < 0.01; −21.6%, *p* < 0.01 or −50.7%, *p* < 0.01) and the corresponding neurobehavioral outcomes were significantly improved in the three groups of EPC-treated mice compared with controls. What is more, AKG-treated diabetic BM-EPCs showed a stronger therapeutic efficacy against cerebral ischemic injury than diabetic BM-EPCs. Furthermore, the angiogenesis in ischemic brain was assessed on day 3 after middle cerebral artery occlusion. It was found that the number of CD31^+^ microvessels in the boundary area of ischemic brains was significantly increased in the three groups of EPC-treated diabetic mice compared with controls (+54.9%, *p* < 0.01; +20.6%, *p* < 0.05 or +62.6%, *p* < 0.01). However, AKG-treated diabetic BM-EPCs exerted a markedly stronger effect on local angiogenesis promotion compared to the diabetic BM-EPCs (+42.0%, *p* < 0.05) (Figure 5F).

These findings collectively demonstrated that the transplanted EPCs could promote local neovascularization capacity and protect against the cerebral ischemic injury in diabetic mice, and the AKG increased the therapeutic effect of EPCs from diabetic mice on angiogenesis promotion and cerebral ischemic injury reduction.

### 3.8 Effects of AKG on HUVECs viability

To check whether the same results observed in EPCs could be obtained in human endothelial cells, we repeated these experiments on HUVECs.

To evaluate the effects of different concentrations of AKG on HUVECs viability, a CCK-8 assay was performed. It was found that cell viability was not affected after incubating with 0.05, 0.1, 0.5, 1, 2 and 8 mM AKG for 24 h. Based on this, 0.05, 0.5 and 1 mM AKG were selected in the functional studies.

### 3.9 AKG reversed the HUVECs dysfunction induced by high glucose

**FIGURE 6:**
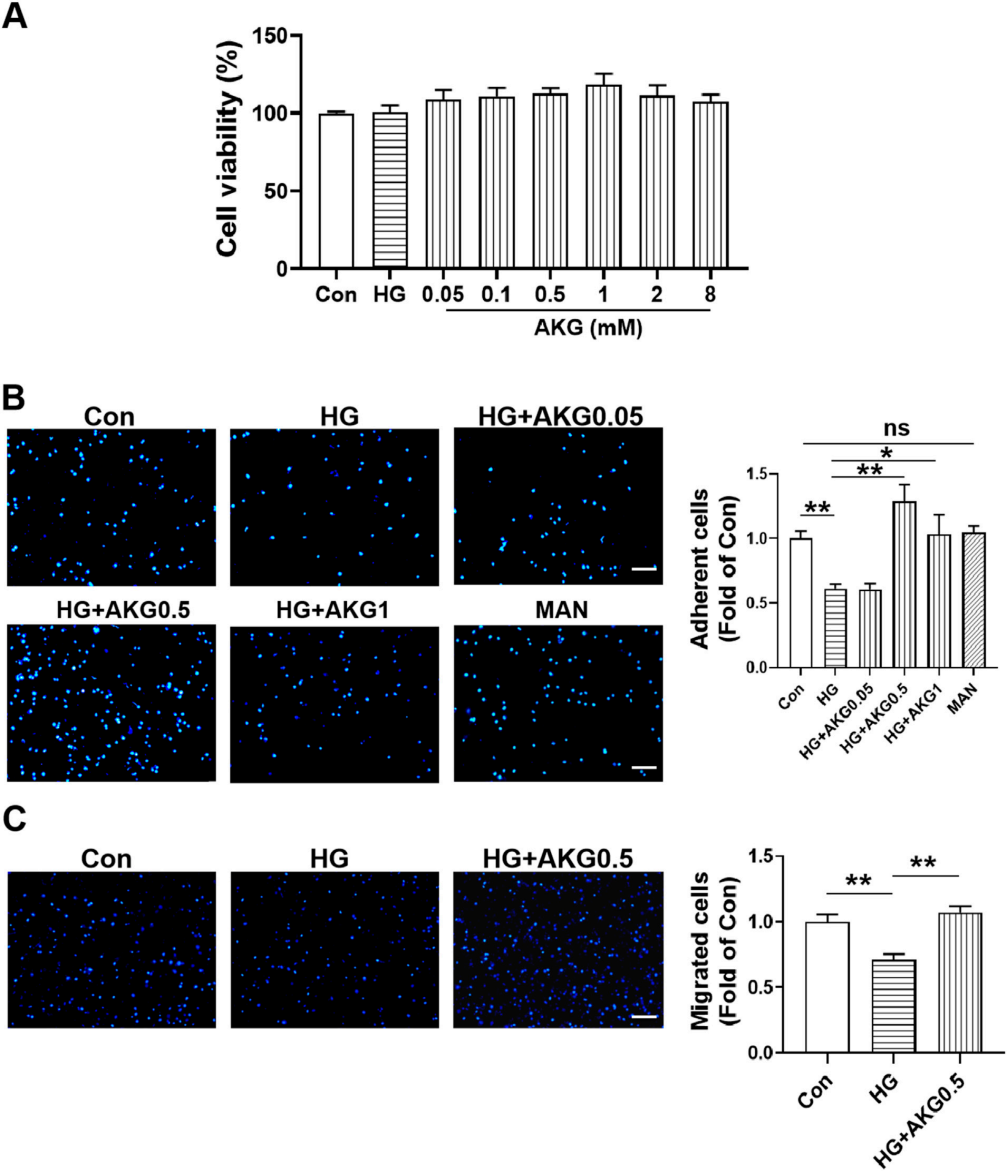
Effects of AKG on cells viability, adhesion and migration functions in HUVECs. **(A)** Effect of high glucose and AKG on cells viability by CCK-8 assay in HUVECs at 24-h (n = 6). **(B)** Cell adhesion was detected by adhesion assay. HUVECs were incubated with vehicle (M199), high glucose (33 mM), high glucose with AKG (0.05, 0.5 and 1 mM) or mannitol respectively for 24 h. Then the adhesion number of cells was evaluated. Representative images (left) and quantification (right) of wound repair at 4-h (n = 5–6). Scale bar: 100 μm. **(C)** Cell migration was detected by transwell migration assay. HUVECs were incubated with high glucose and AKG (0.5 mM) for 24-h. Then the migration number of cells was evaluated. Representative images (left) and quantification (right) of migrated cells at 24-h (n = 6). Scale bar: 100 μm. AKG, Alpha-ketoglutarate. Data are presented as means ± S.E.M. *p < 0.05, **p < 0.01.

To determine whether AKG exerted direct beneficial effects in protecting HUVECs functions, we further performed cell functions, HUVECs were treated with vehicle (M199), high glucose (33 mM) or high glucose with additional AKG (0.05, 0.5 and 1 mM) respectively for 1 day. Equimolar mannitol addition controlled for any potential effect of increased osmolarity under high-glucose conditions. It was found the adhesion function was significantly impaired in high-glucose-treated HUVECs, which was rescued by AKG treatment (0.5 mM for 1 day, *p* < 0.01, 1 mM for 1 day, *p* < 0.05), but was not affected after incubating with 0.05 mM AKG (Figure 6B). As a control for potential osmotic effects of added high glucose, equimolar mannitol produced no impairment of adhesion function in HUVECs (Figure 6B). Hence, we next selected the concentration of 0.5 mM for the subsequent *in vitro* experiments. Transwell migration assay showed that the migration number was also significantly increased after treating with 0.5 mM AKG compared with HG (0.5 mM for 1 day, *p* < 0.01) (Figure 6C).

### 3.10 AKG increased the levels of MnSOD and CuZnSOD in HUVECs exposed to high glucose

**FIGURE 7:**
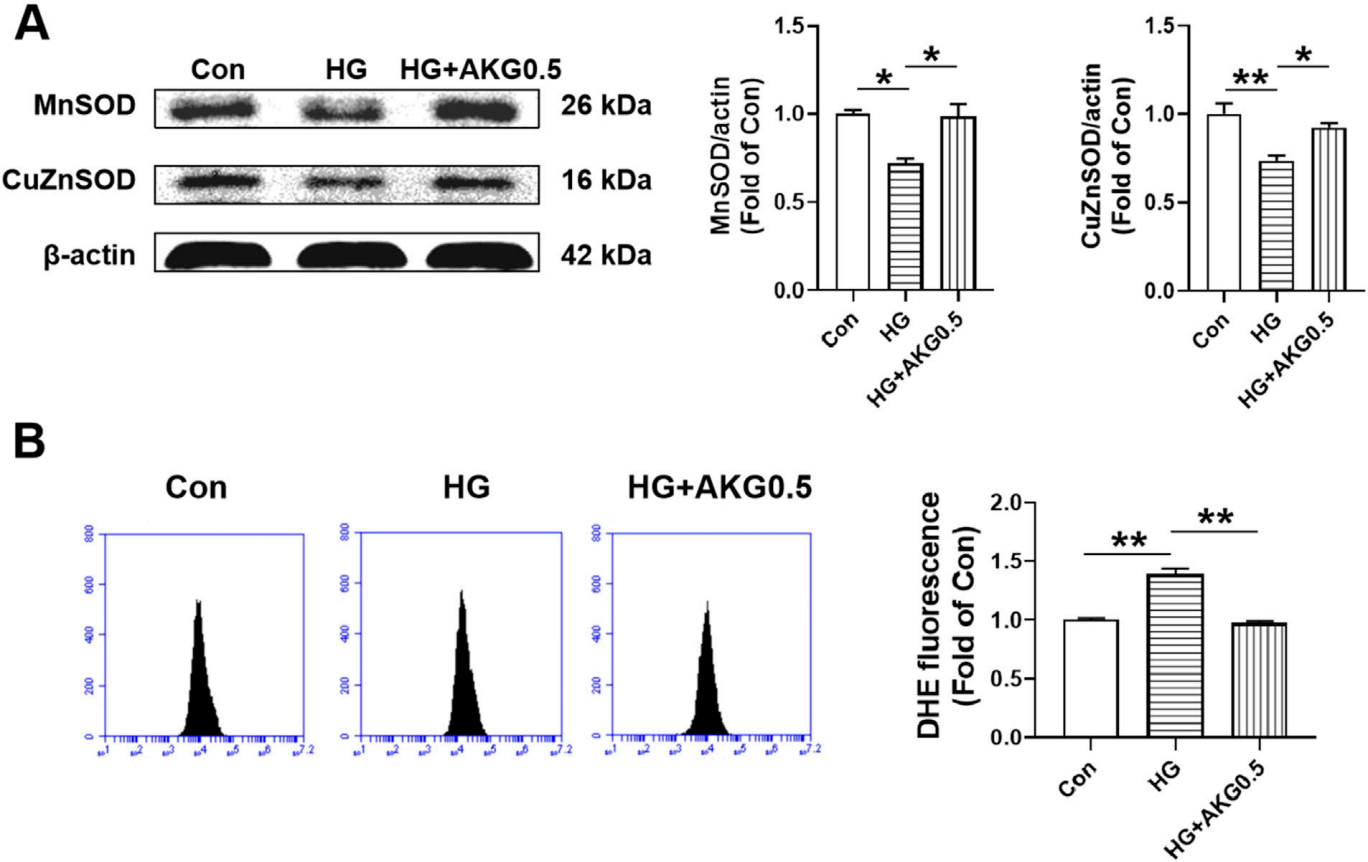
Effects of AKG administration on MnSOD, CuZnSOD and intracellular O_2_^·-^ levels of HUVECs in high glucose (33 mM). HUVECs were treated with vehicle (M199), high glucose (33 mM and high glucose with AKG (0.5 mΜ) and 24 h later, the cells functions, intracellular O_2_^·-^ level and MnSOD and CuZnSOD expression levels were examined. **(A)** The representative images and the protein expression levels of MnSOD (n = 3) and CuZnSOD (n = 3). **(B)** Intracellular O_2_^·-^ levels of HUVECs (n = 6). *p < 0.05, **p < 0.01. Con, vehicle (M199)-treated HUVECs; HG, high glucose-treated HUVECs; HG + AKG0.5, HUVECs treated with high glucose and additional AKG (0.5 mΜ, 24 h); AKG, Alpha-ketoglutarate.

Since SOD is an important antioxidant enzyme which can clear the O_2_^·-^ and protect cells from oxidative damage, the expression levels of MnSOD and CuZnSOD were assessed. Compared to control, both MnSOD and CuZnSOD were significantly decreased in HUVECs in HG (−28.0%, *p* < 0.05; −26.5%, *p* < 0.01), while AKG increase the expression levels of MnSOD and CuZnSOD from HG (+26.2%, *p* < 0.05; +19.0%, *p* < 0.05). These results suggest that the increase of MnSOD and CuZnSOD may contribute to the therapy mitigated the adhesion incapacity of HUVECs in high glucose by AKG (Figure 7A). As shown in Figure 7B, intracellular O_2_^·-^ level of HUVECs from HG was increased ∼40% compared to control group (+38.6%, *p* < 0.01). However, it was significantly attenuated in HUVECs from AKG treated when compared with HG (−41.6%, *p* < 0.01).

### 3.11 AKG inhibited pro-inflammatory cytokine production via TLR4 signaling pathway in HUVECs

**FIGURE 8:**
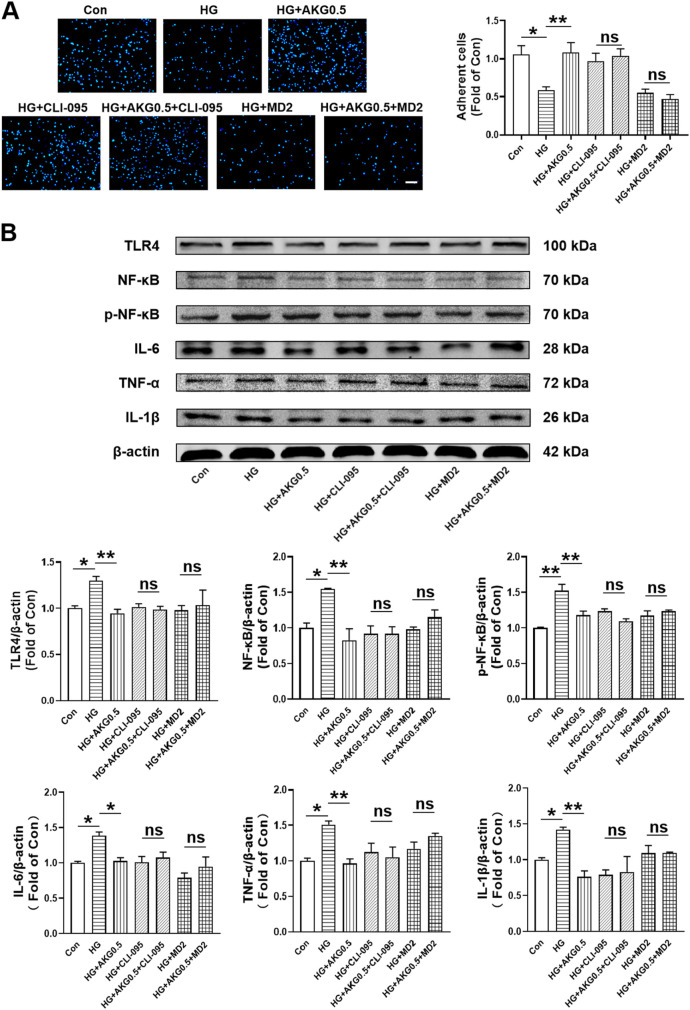
Inhibiting TLR4 abolished the improvement of HUVEC functions induced by AKG treatment. HUVECs were pretreated with or without TLR4 inhibitors (CLI095, 10 μM; MD2-TLR-4-IN-1, 10 µM) for 1-h before co-incubation with high glucose (33 mM) and/or AKG (0.5 mM) for 24-h, then the cells functions, TLR4, NF-κB, p-NF-κB, IL-6, TNF-α and IL-1β expression levels were examined. **(A)** Adhesion assay of HUVECs (n = 5–6). Scale bar, 100 μm. **(B)** The representative images and the protein expression levels of TLR4 (n = 4–5), NF-κB (n = 3), p-NF-κB (n = 3), IL-6 (n = 4), TNF-α (n = 3) and IL-1β (n = 3–4). *p < 0.05, **p < 0.01. Con, vehicle (M199)-treated HUVECs; HG, high glucose-treated HUVECs; HG + AKG0.5, HUVECs treated with high glucose and additional AKG (0.5 mΜ, 24 h); HG + CLI-095, HUVECs treated with high glucose and CLI-095 (10 μM, 1 h); HG + AKG0.5+CLI-095, HUVECs treated with high glucose with AKG (0.5 mΜ, 24 h) and CLI-095 (10 μM, 1 h); HG + MD2, HUVECs treated with high glucose and MD2-TLR-4-IN-1 (10 μM, 1 h); HG + AKG0.5+MD2, HUVECs treated with high glucose with AKG (0.5 mΜ, 24 h) and MD2-TLR-4-IN-1 (10 μM, 1 h); AKG, Alpha-ketoglutarate.

To more thoroughly assess whether AKG suppressed inflammatory responses through TLR4, TLR4 signaling inhibitors (CLI095 and MD2-TLR-4-IN-1) were incubated with HUVECs. Cell adhesion assay showed that compare with HG, CLI-095 can increase the number of adherent cells (+38.6%, *p* < 0.01). AKG-induced adhesion function improvement vanished after pretreatment with the TLR4 signaling inhibitor CLI-095 in high glucose-incubated HUVECs (Figure 8A). MD2-TLR-4-IN-1 does not affect the number of adherent cells. AKG-induced adhesion function improvement also disappeared after pretreatment with the TLR4 signaling inhibitor MD2-TLR-4-IN-1 in high glucose-incubated HUVECs (Figure 8A). The expression levels of TLR4, NF-κB, p-NF-κB, IL-6, TNF-α and IL-1β, were examined through Western blotting. Comparing HG to the Con (+29.9%, *p* < 0.05; +54.7%, *p* < 0.05; +52.3%, *p* < 0.01; +38.4%, *p* < 0.05; +50.7%, *p* < 0.05 and +41.9%, *p* < 0.05 respectively), there was a substantial rise in the expression of TLR4, NF-κB, p-NF-κB, IL-6, TNF-α and IL-1β intracellular levels, while AKG remarkably attenuated these effects (−35.2%, *p* < 0.01; −72.2%, *p* < 0.01; −34.5%, *p* < 0.01; −35.6%, *p* < 0.05; −53.9%, *p* < 0.01 and −65.7%, *p* < 0.01 respectively) (Figure 8B). These studies showed that, in HG-stimulated HUVECs, AKG reduced the expression of TLR4 and pro-inflammatory cytokines. AKG’s effect on the inflammatory cytokine also disappeared after pretreatment with the TLR4 signaling inhibitor MD2-TLR-4-IN-1 in high glucose-incubated HUVECs. These findings imply that TLR4 may contribute to AKG’s ability to reduce HG-induced inflammation in HUVECs.

### 3.12 AKG inhibited pro-inflammatory cytokine production via NF-κB signaling pathway in HUVECs

**FIGURE 9:**
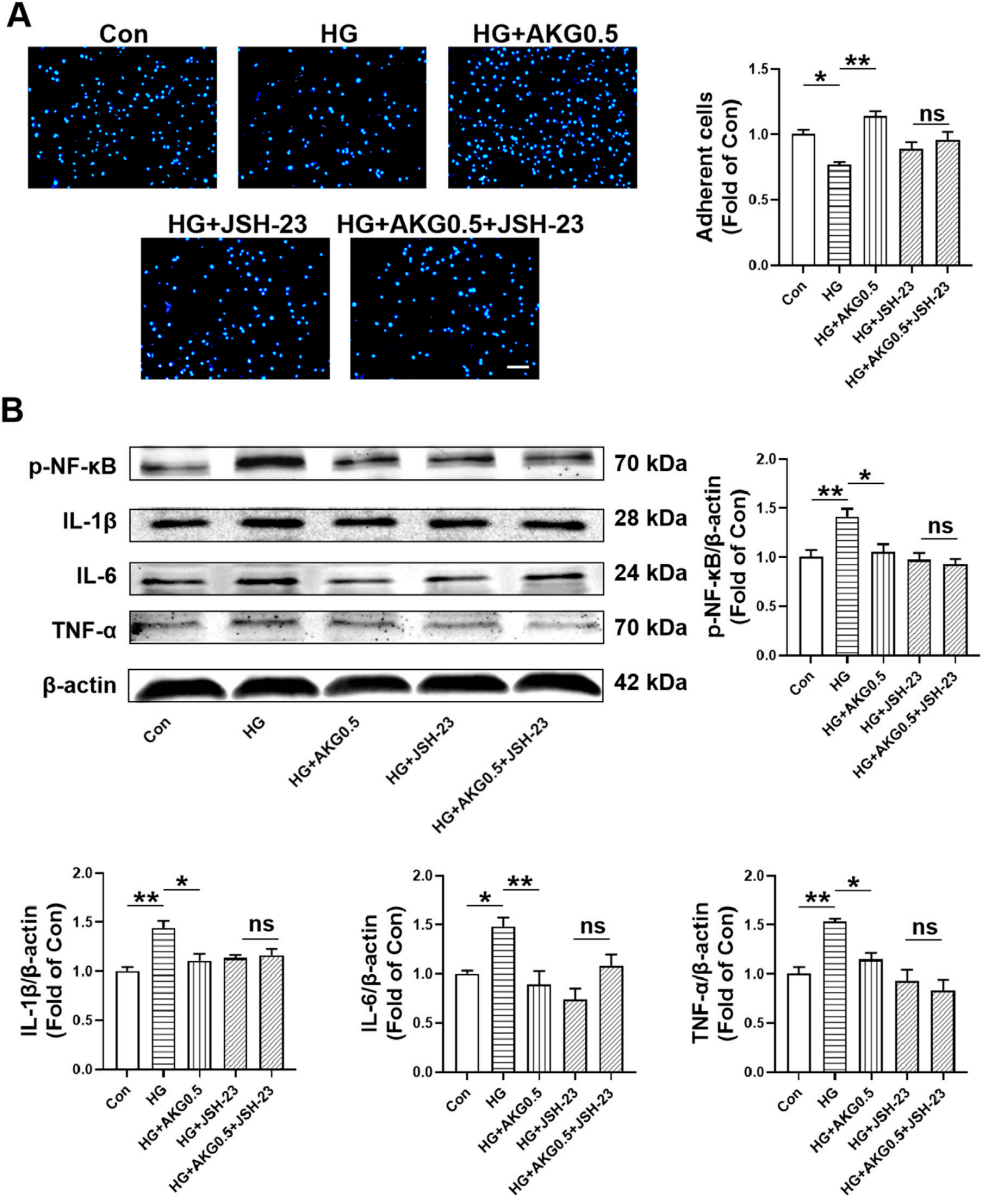
Inhibiting NF-κB abolished the improvement of HUVEC functions induced by AKG treatment. HUVECs were pretreated with or without NF-κB inhibitors (JSH-23, 10 µM) for 1-h before co-incubation with high glucose (33 mM) and/or AKG (0.5 mM) for 24-h, then the cells functions, p-NF-κB, IL-6, TNF-α and IL-1β expression levels were examined. **(A)** Adhesion assay of HUVECs (n = 10–12). Scale bar, 100 μm. **(B)** The representative images and the protein expression levels of p-NF-κB (n = 4), IL-6 (n = 4), TNF-α (n = 4) and IL-1β (n = 3). *p < 0.05, **p < 0.01. Con, vehicle (M199)-treated HUVECs; HG, high glucose-treated HUVECs; HG + AKG0.5, HUVECs treated with high glucose and additional AKG (0.5 mΜ, 24 h); HG + JSH-23, HUVECs treated with high glucose and JSH-23 (10 μM, 1 h); HG + AKG0.5+JSH-23, HUVECs treated with high glucose with AKG (0.5 mΜ, 24 h) and JSH-23 (10 μM, 1 h); AKG, Alpha-ketoglutarate.

To further investigate whether AKG inhibited pro-inflammatory cytokine production via NF-κB signaling pathway, NF-κB signaling inhibitors (JSH-23) were incubated with HUVECs. AKG-induced adhesion functional improvement vanished after pretreatment with the NF-κB signaling inhibitor JSH-23 in high glucose-incubated HUVECs (*p* < 0.05; Figure 9A). The expression levels of p-NF-κB, IL-1β, IL-6 and TNF-α, were examined through Western blotting. Comparing HG to the Con, there was a substantial rise in the expression of p-NF-κB, IL-1β, IL-6 and TNF-α intracellular levels (+41.3%, *p* < 0.01; +44.2%, *p* < 0.01; +48.2%, *p* < 0.05 and +53.1%, *p* < 0.01, respectively), while AKG remarkably attenuated these effects (−35.7%, *p* < 0.05; −33.5%, *p* < 0.05; −58.7%, *p* < 0.01 and −38.2%, *p* < 0.05) (Figure 9B). These studies showed that, in HG-stimulated HUVECs, AKG reduced the expression of p-NF-κB, IL-1β, IL-6 and TNF-α. Treatment with inhibitors of NF-κB signal transduction (JSH-23) and AKG all suppressed HG-induced overexpression of p-NF-κB, IL-1β, IL-6 and TNF-α in HUVECs (*p* < 0.05 and *p* < 0.01). Additionally, this protective effect disappeared after NF-κB signaling inhibitor JSH-23 addition. These findings imply that NF-κB may contribute to AKG’s ability to reduce HG-induced inflammation in HUVECs.

## 4 Discussion

The major findings of the present study are as follows: (1) AKG rescued impaired EPC-mediated angiogenesis and prevented against cerebral ischemic injury in diabetic mice; (2) AKG treatment increased the expression of MnSOD and CuZnSOD and decrease the intracellular O_2_^·-^ in EPCs from diabetic mice; (3) AKG treatment decreased the expression of proinflammatory factors TLR4, NF-κB, IL-6 and IL-1β in EPCs from diabetic mice; (4) AKG rescued the functions of high glucose-stimulated HUVECs by reducing inflammation through the TLR4/NF-κB pathway and attenuating oxidative stress. This study assessed the effects of AKG on angiogenesis and neurological function on day 3 post-cerebral ischemia, reflecting only acute-phase outcomes and failing to address long-term vascular remodeling and neural repair. The diabetic microenvironment may delay chronic repair processes, limiting the comprehensiveness of the findings. Future studies would extend observation periods to 1–6 months to evaluate the long-term effects of AKG on vascular density, neurological function, and neurogenesis, and employ dynamic monitoring and diabetes-specific experiments to validate its glucose-independent protective mechanisms. Since AKG has long been used as a nutritional supplement in athletes to increase the size and strength of their muscles, its safety has been proved (Guo et al., 2022). Furthermore, conducting phase I/II clinical trials to evaluate the safety, pharmacokinetics, and effects of AKG on cardiovascular and peripheral vascular function in diabetic patients will significantly advance its clinical translation and applicability across multiple organ ischemic complications.

It has been shown before that intravenous ornithine AKG, given at a dose of 25 g on 5 consecutive days, significantly improves the recovery of neurological function in patients with recent stroke (Woollard et al., 1978). According to the conversion ratio between mice and humans (9.1), the daily AKG dose (g/kg) for mice can be calculated as the following formula: conversion ratio of surface area between mice and humans (9.1) × a daily dose of an adult (25 g)/average weight of adults (60 kg), that is: 9.1 × 25 (g)/60 (kg) = 4 g/kg. Furthermore, supplementation with 2% AKG in standard chow and water for 9 weeks has been shown to ameliorate chronic cardiac dysfunction induced by pressure overload in mice (An et al., 2021). Similarly, dietary supplementation with 2% calcium AKG (CaAKG) extends lifespan and reduces morbidity in aging mice (Asadi Shahmirzadi et al., 2020). In male C57BL/6 mice fed a high-fat diet, supplementation with 2% AKG in drinking water significantly reduced circulating glucose and glycated hemoglobin levels while increasing insulin levels (Yuan et al., 2022). To ensure consistency in AKG intake among mice, we conducted a preliminary experiment to measure water intake. Based on daily water consumption, AKG at 2% in drinking water corresponds to doses of 4 g·kg^−1^·d^−1^. However, this study evaluated only a single AKG dose (4 g·kg^−1^·d^−1^), without dose-response analysis, limiting insights into the optimal therapeutic window. Moreover, high doses may induce off-target effects, and the translatability of mouse doses to human equivalent doses remains unverified. Future studies would test multiple AKG doses (2–8 g·kg^−1^·d^−1^) to assess dose-dependent effects on EPC function and angiogenesis, optimize dosing through pharmacokinetic analysis, and employ transcriptomics to investigate off-target effects, thereby enhancing AKG’s clinical applicability. In future studies, we will assess AKG’s safety through serum biochemical, histological, and pharmacokinetic analyses, investigate its impact on non-target pathways and the gut microbiome, and validate its chronic safety in long-term administration experiments.

A previous study reveals that AKG supplementation (3.80 ± 0.44 g/kg body weight per day) increased the average food intake but reduced the body weight gain rate in mice (Chen et al., 2017). It has shown that 1% AKG supplementation in the basal diet had little effect on feed intake and weight gain in LPS-challenged piglets (Hou et al., 2010). Similarly, a study reported that AKG (1% in drinking water) did not significantly alter food intake in mice fed a high-fat high-fructose diet (Vatashchuk et al., 2025). The effects of AKG supplementation on body weight and feed intake differ among those investigations. This discrepancy may be related to species, disease models, methodological differences or the dosage of AKG supplementation in these studies.

It has been shown that long-term systemic treatment of AKG effectively lowered blood glucose levels, which were associated with decreased hepatic gluconeogenesis and increased insulin secretion, in diet-induced obesity and *db/db* mice. Similarly, a study reported that AKG (1% in drinking water) did not significantly alter food intake in mice fed a high-fat high-fructose diet but improved metabolic parameters, such as glucose tolerance (Vatashchuk et al., 2025). Notably, the inhibitory effects of AKG on glycemia and hepatic gluconeogenesis were preserved in STZ-treated type 1 diabetes mice (Yuan et al., 2022). Here, we observed that AKG treatment slightly reduced the blood glucose levels in STZ-treated type 1 diabetes mice, but the differences were not statistically significant.

Diabetes have been reported to aggravate damage following cerebrovascular disorder owing to the involvement of many deleterious pathways including oxidative stress, impaired leukocyte function, abnormal angiogenesis, increased blood-brain barrier (BBB) permeability, and inflammatory responses (Shukla et al., 2017). Angiogenesis is the fundamental processes during formation of new blood vessels after vascular injury, following ischemic stroke (Liman and Endres, 2012). EPCs, as important precursors of endothelial cells, have been shown to participate in vascular formation, and have been used to successfully restore endothelial function and enhance neovascularization capacity in ischemic brain (Marrotte et al., 2010; Simpkins et al., 1997). Here we found, for the first time, that treatment with AKG significantly improve EPC function, enhance angiogenesis in the ischemic brain and reduce the cerebral ischemic injury in diabetic mice. Moreover, the present work demonstrated that the transplanted EPCs could incorporate into the area of ischemic boundary, promote local neovascularization capacity and protect against the cerebral ischemic injury in diabetic mice, and the AKG increased the therapeutic effect of EPCs from diabetic mice on angiogenesis promotion and cerebral ischemic injury reduction. Taken together, these findings demonstrate that the promotion of EPC-mediated ischemic brain angiogenesis may partly contribute to the protection of AKG against cerebral ischemic injury in diabetic mice. In addition, it has been demonstrated that AKG promotes cardiac regeneration post-myocardial infarction by inducing cardiomyocyte proliferation (Shi et al., 2024), and EPCs participate in vascular repair and revascularization in various ischemic organs besides brain tissue (Marrotte et al., 2010; Sugiura et al., 2008). Thus, AKG treatment might also be a strategy to protect against other diabetic cardiovascular complications except cerebral ischemia, such as myocardial infarction and amputation, which remains to be test by further studies.

It has been shown that MnSOD and CuZnSOD critically regulate EPC function (Zhang et al., 2002; Marrotte et al., 2010; Hamed et al., 2009). O_2_·^-^ play important roles in the pathogenesis of many cardiovascular diseases, including hypertension and atherosclerosis. The increase in intracellular O_2_^·-^ represent a major mechanism underlying EPC dysfunction in diabetic and hypertensive mice (An et al., 2021; Zhu et al., 2008; Simpkins et al., 1997). SODs are the major antioxidant defense systems against O_2_·^-^ (Fukai and Ushio-Fukai, 2011). Thus, AKG treatment increased the MnSOD and CuZnSOD levels with the decrease intracellular O_2_·^-^ levels, as observed in the present study, might partly contribute to the promotion of EPC functions in AKG-treated diabetic mice.

Endothelial inflammatory responses mediated by TLR4 are involved in the progression of atherosclerosis associated with diabetes mellitus (Wada and Makino, 2016). Induced expression of the proinflammatory cytokines and adhesion molecules is a critical step and hallmark of endothelial proinflammatory activation. Nuclear factor (NF)-κB is a master regulator of proinflammatory responses in ECs as well as in other types of cells (Xiao et al., 2014). Recently, it has been shown that AKG exhibits an anti-inflammatory effect (Zdzisińska et al., 2017; Bayliak and Lushchak, 2021; Jia et al., 2023). Our results demonstrated that the expression levels of TLR4, NF-κB, IL-6 and IL-1β were markedly increased in diabetic EPCs, which could be decreased by AKG treatment. Thus, AKG probably alleviates the impairment of diabetic EPCs by downregulating inflammation via TLR4/NF-κB signaling pathway. In future studies, we will investigate the role of the TLR4/NF-κB signaling cascade in AKG’s anti-inflammatory responses, using *in vivo* genetic or pharmacological inhibition. Additionally, AKG may modulate NF-κB in parallel through Nrf2 or c-Fos/IL-10 pathways (Cheng et al., 2024; Hua et al., 2022). Future studies could validate AKG’s protective effects in TLR4^−/−^ or NF-κB p65^−/−^ mice, employ pharmacological inhibition to confirm pathway specificity, and utilize transcriptomics and epigenetics analyses to investigate the potential roles of c-Fos, Nrf2, or TET enzymes as upstream regulatory factors.

To check whether the same results observed in EPCs could be obtained in human endothelial cells, we repeated these experiments on HUVECs. We found that AKG significantly inhibited high glucose-induced overexpression of TLR4, p-NF-κB, NF-κB, IL-6, TNF-α, and IL-1β in HUVECs. Moreover, treatment with TLR4 or NF-κB inhibitors weaken the effect of AKG on inflammation in high glucose-incubated HUVECs. In addition, we demonstrated that TLR4 or NF-κB inhibitors weakened the adhesion function improvement caused by AKG treatment in high glucose-incubated HUVECs. These results suggest that the TLR4/NF-κB signaling pathway may be involved in the mechanism by which AKG inhibits high glucose-induced inflammation in HUVECs. However, the present work is limited to murine models and HUVECs, and inclusion of human data or validation in future studies would greatly enhance clinical relevance.

**FIGURE 10:**
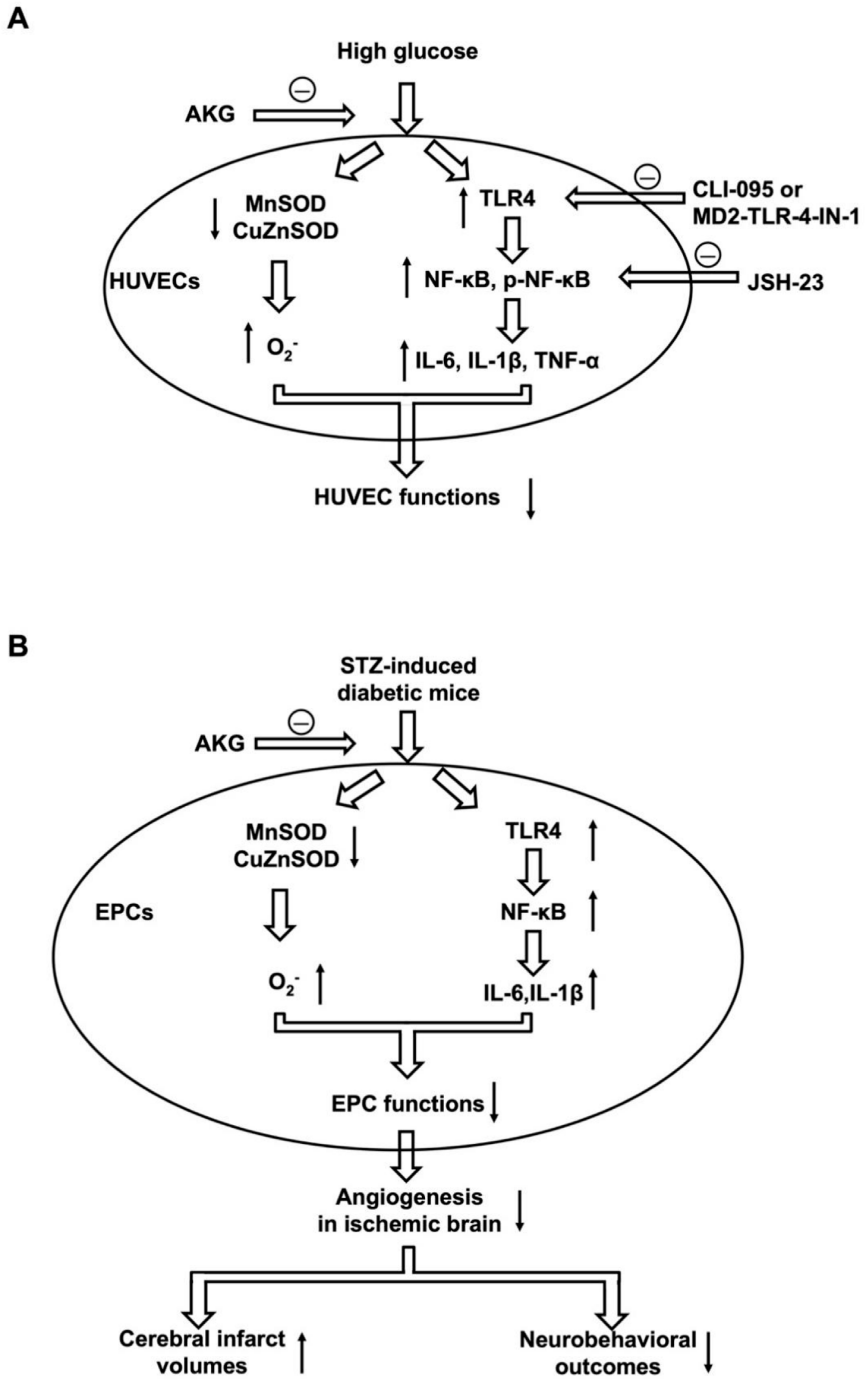
Putative mechanisms underlying AKG reducing cerebral ischemic injury in diabetic mice. **(A)** The decreased expression of MnSOD and CuZnSOD, increased intracellular O_2_^·-^ levels, and increased expression of TLR4, NF-κB, p-NF-κB, IL-6, IL-1β and TNF-α contribute to impaired HUVEC functions under high glucose conditions, which is rescued by AKG treatment. **(B)** The decreased expression of MnSOD and CuZnSOD, increased intracellular O_2_^·-^ levels, and increased expression of TLR4, NF-κB, IL-6 and IL-1β contribute to impaired EPC functions in diabetes mice. Diabetic mice have impaired EPC function, decreased ischemic angiogenesis, increased cerebral infarct volumes, and reduced corresponding neurobehavioral outcome. AKG reduces cerebral ischemic injury via rescuing impaired EPC-mediated angiogenesis in diabetic mice. AKG, Alpha-ketoglutarate; HUVEC, human umbilical vein endothelial cells; CLI095, TLR4 inhibitor; MD2-TLR-4-IN-1, TLR4 inhibitor; JSH-23, NF-κB inhibitor; STZ, streptozotocin; EPC, endothelial progenitor cell.

In conclusion, the present study demonstrated that AKG could prevent cerebral ischemic injury in diabetic mice, which might be partly attributed to the improvement of EPC-mediated ischemic brain angiogenesis (Figure 10). This result implies that AKG treatment may be a safe and effective manner to prevent ischemic diseases (including stroke) in diabetes.

## Data Availability

The raw data supporting the conclusions of this article will be made available by the authors, without undue reservation.
